# Sensitivity and specificity of a real-time reverse transcriptase polymerase chain reaction detecting feline coronavirus mutations in effusion and serum/plasma of cats to diagnose feline infectious peritonitis

**DOI:** 10.1186/s12917-017-1147-8

**Published:** 2017-08-02

**Authors:** Sandra Felten, Christian M. Leutenegger, Hans-Joerg Balzer, Nikola Pantchev, Kaspar Matiasek, Gerhard Wess, Herman Egberink, Katrin Hartmann

**Affiliations:** 1 Clinic of Small Animal Medicine, Centre for Clinical Veterinary Medicine, Ludwig-Maximilians-Universitaet Munich, Veterinaerstr. 13, 80539 Munich, Germany; 2 IDEXX Laboratories, Inc., 2825 KOVR Drive, West Sacramento, USA; 3 IDEXX Laboratories, Moerikestr. 28/3, 71636 Ludwigsburg, Germany; 4 Section of Clinical & Comparative Neuropathology, Centre for Clinical Veterinary Medicine, Ludwig-Maximilians-Universitaet Munich, Veterinaerstr. 13, 80539 Munich, Germany; 5 Faculty of Veterinary Medicine, Department of Infectious Diseases and Immunology, Utrecht University, Yalelaan 1, 3584 CL Utrecht, The Netherlands

**Keywords:** Feline infectious peritonitis (FIP), Reverse transcriptase polymerase chain reaction (RT-PCR), Feline coronavirus (FCoV), Feline enteric coronavirus (FECV), Feline infectious peritonitis virus (FIPV)

## Abstract

**Background:**

Feline coronavirus (FCoV) exists as two pathotypes, and FCoV spike gene mutations are considered responsible for the pathotypic switch in feline infectious peritonitis (FIP) pathogenesis. The aim of this study was to evaluate sensitivity and specificity of a real-time reverse transcriptase polymerase chain reaction (RT-PCR) specifically designed to detect FCoV spike gene mutations at two nucleotide positions. It was hypothesized that this test would correctly discriminate feline infectious peritonitis virus (FIPV) and feline enteric coronavirus (FECV).

**Methods:**

The study included 63 cats with signs consistent with FIP. FIP was confirmed in 38 cats. Twenty-five control cats were definitively diagnosed with a disease other than FIP. Effusion and/or serum/plasma samples were examined by real-time RT-PCR targeting the two FCoV spike gene fusion peptide mutations M1058 L and S1060A using an allelic discrimination approach. Sensitivity, specificity, negative and positive predictive values including 95% confidence intervals (95% CI) were calculated.

**Results:**

FIPV was detected in the effusion of 25/59 cats, one of them being a control cat with chronic kidney disease. A mixed population of FIPV/FECV was detected in the effusion of 2/59 cats; all of them had FIP. RT-PCR was negative or the pathotype could not be determined in 34/59 effusion samples. In effusion, sensitivity was 68.6% (95% CI 50.7–83.2), specificity was 95.8% (95% CI 78.9–99.9). No serum/plasma samples were positive for FIPV.

**Conclusions:**

Although specificity of the test in effusions was high, one false positive result occurred. The use of serum/plasma cannot be recommended due to a low viral load in blood.

## Background

The key event in the pathogenesis of feline infectious peritonitis (FIP) is the switch in viral cell tropism, which originates from mutations of the feline coronavirus (FCoV) genome [1]. According to the internal mutation hypothesis, feline infectious peritonitis virus (FIPV) emerges from feline enteric coronavirus (FECV) by spontaneous mutations within an infected cat [2, 3]. While FECV causes asymptomatic infection or mild enteritis and is widespread among the cat population [4–6] and especially common in multi-cat environments [7–9], FIPV causes FIP, a lethal immune-mediated disease [10, 11]. FECV has a tropism for the small intestine apical villi epithelium [4, 12], while FIPV can infect monocytes/macrophages and replicate sufficiently within these cells to allow systemic spread and macrophage activation [13].

Mutations in the FCoV spike gene and resulting amino acid substitutions in the spike protein are considered responsible for the acquisition of macrophage tropism due to the spike protein’s role in receptor binding and cell entry [14–16]. Although amino acid substitutions M1058L and S1060A within the spike protein correlated with the FIP phenotype in >95% of cases in one study [15], a subsequent study found them to be rather associated with systemic spread of FCoV in cats with and without FIP [14]. Focusing on a furin cleavage site in the region between receptor-binding and fusion domains of the spike gene, a recent study detected functionally relevant mutations strongly correlated with FIP and documented the emergence of one of these substitutions in a cat during the development of FIP [16].

Definitive ante-mortem diagnosis currently still requires invasive tissue sample collection for immunohistochemical demonstration of FCoV antigen in macrophages in tissue lesions [17–20]. Reverse transcriptase polymerase chain reaction (RT-PCR) is frequently applied to detect FCoV RNA in diagnostic samples, and recent studies reported relatively satisfying results for real-time RT-PCR results using different materials [21–23]. Nevertheless, standard RT-PCR cannot distinguish FECV from FIPV and has been shown to detect FCoV RNA also in the blood of healthy cats that never developed FIP [24, 25].

Therefore, it was the aim of this study to evaluate sensitivity and specificity of a real-time RT-PCR (FIP Virus RealPCR Test, IDEXX Laboratories) able to discriminate between FECV and FIPV in effusions and serum/plasma. It was hypothesized that this discriminative PCR would correctly identify FIPV and thus, would be a non-invasive and reliable method to definitively diagnose FIP.

## Methods

### Animals

Overall, 63 cats with signs consistent with FIP were included in the study. All cats were presented either as sick feline patients (*n* = 48) or directly submitted for necropsy (*n* = 15).

**Fig. 1:**
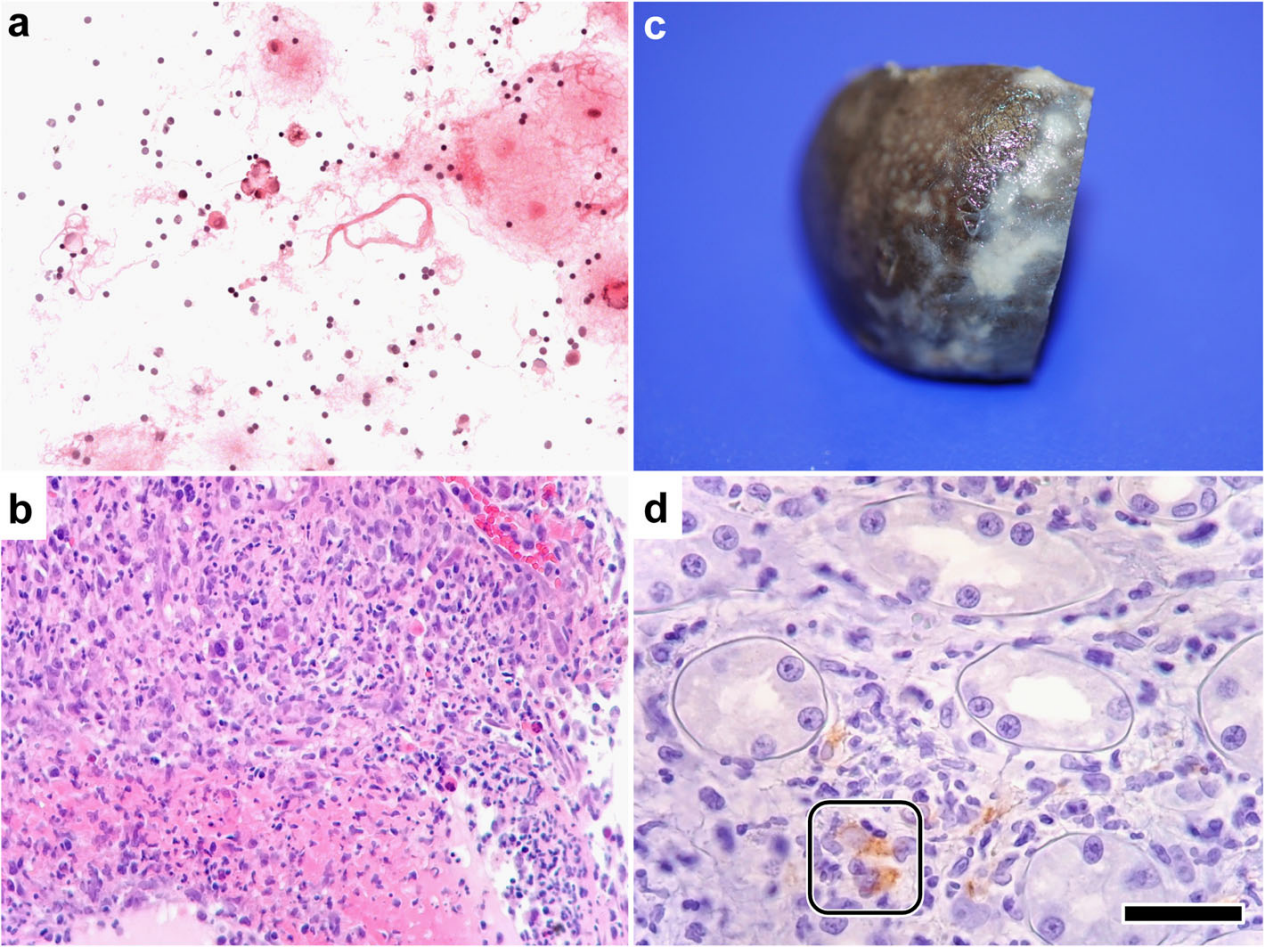
Morphological and immunohistochemical diagnosis of feline infectious peritonitis (FIP) featuring typical effusion cytology (**a**), subcapsular changes in visceral organs (**b**; liver), inflammatory and fibrinonecrotic changes (**c**) and macrophages immunopositive for feline coronavirus (FCoV) antigen (**d**). Staining: a,c: haematoxylin-eosin; d: haematoxylin. Scale bar: 75 μm for a and c; 1 cm for b, 25 μm for d

For 38 cats (FIP group, Table 1), a definitive diagnosis of FIP was established post-mortem either by histopathology (*n* = 10) (Fig. 1a-c), or by histopathology and immunohistochemical (IHC) staining of FCoV antigen in tissue samples (Fig. 1d) obtained at necropsy (*n* = 28). In the cats with histopathological confirmation, a diagnosis of FIP was based on the occurrence of effusions (Fig. 1a) and/or yellow to white foci or nodules in different organs (Fig. 1b) plus presence of typical histological lesions, including plasma-cellular perivasculitis and/or accumulation of plasma cells accompanied by a necro-purulent inflammation (Fig. 1c).

**Table 1:** Detailed information for cats of the feline infectious peritonitis (FIP) group

cat	symptoms leading to inclusion	diagnosis	method of confirmation of diagnosis	samples available	result of RT-PCR of effusion samples	result of RT-PCR of serum/plasma samples	detected mutation
1	pleural effusion, fever, uveitis	FIP	histopathology plus immunohistochemistry	effusion	FIPV	n.d.	M1058L
2	pleural effusion	FIP	histopathology plus immunohistochemistry	effusion	negative	n.d.	n.d.
3	ascites, icterus	FIP	histopathology plus immunohistochemistry	effusion	FIPV	n.d.	M1058L
4	ascites, icterus	FIP	histopathology plus immunohistochemistry	effusion	FIPV	n.d.	M1058L
5	ascites, fever, icterus	FIP	histopathology plus immunohistochemistry	effusion	FIPV	n.d.	M1058L
6	ascites, fever, icterus	FIP	histopathology plus immunohistochemistry	effusion	mixed	n.d.	n.d.
7	ascites, icterus	FIP	histopathology plus immunohistochemistry	effusion	FIPV	n.d.	M1058L
8	ascites, neurological signs, uveitis	FIP	histopathology plus immunohistochemistry	effusion	FIPV	n.d.	M1058L
9	ascites	FIP	histopathology plus immunohistochemistry	effusion	BLD	n.d.	n.d.
10	ascites	FIP	histopathology plus immunohistochemistry	effusion	BLD	n.d.	n.d.
11	ascites, icterus, hyperglobulinemia	FIP	histopathology plus immunohistochemistry	effusion and plasma	FIPV	BLD	M1058L
12	fever, icterus, neurological signs, hyperglobulinemia	FIP	histopathology plus immunohistochemistry	plasma	n.d.	negative	n.d.
13	ascites, icterus	FIP	histopathology plus immunohistochemistry	effusion	FIPV	n.d.	M1058L
14	ascites	FIP	histopathology plus immunohistochemistry	effusion	FIPV	n.d.	M1058L
15	ascites, fever, icterus	FIP	histopathology plus immunohistochemistry	effusion and plasma	FIPV	negative	M1058L
16	ascites, fever, hyperglobulinemia, uveitis	FIP	histopathology plus immunohistochemistry	effusion and plasma	FIPV	negative	M1058L
17	ascites, icterus, neurological signs,hyperglobulinemia	FIP	histopathology plus immunohistochemistry	effusion	FIPV	n.d.	M1058L
18	ascites, fever, icterus	FIP	histopathology plus immunohistochemistry	effusion	FIPV	n.d.	M1058L
19	ascites, fever, hyperglobulinemia	FIP	histopathology plus immunohistochemistry	plasma	n.d.	negative	n.d.
20	pleural effusion, fever, hyperglobulinemia	FIP	histopathology plus immunohistochemistry	effusion and plasma	FIPV	negative	M1058L
21	ascites	FIP	histopathology plus immunohistochemistry	effusion and plasma	mixed	negative	n.d.
22	ascites	FIP	histopathology plus immunohistochemistry	effusion	BLD	n.d.	n.d.
23	ascites	FIP	histopathology plus immunohistochemistry	effusion and plasma	IND	negative	n.d.
24	ascites, icterus	FIP	histopathology plus immunohistochemistry	effusion and plasma	FIPV	negative	M1058L
25	ascites, icterus, hyperglobulinemia	FIP	histopathology plus immunohistochemistry	effusion	FIPV	n.d.	M1058L
26	ascites, icterus	FIP	histopathology plus immunohistochemistry	effusion	FIPV	n.d.	M1058L
27	ascites, icterus, hyperglobulinemia	FIP	histopathology plus immunohistochemistry	effusion	FIPV	n.d.	M1058L
28	ascites and pleural effusion, icterus	FIP	histopathology	serum	n.d.	negative	n.d.
29	ascites, fever, hyperglobulinemia, uveitis	FIP	histopathology	effusion and serum	BLD	negative	n.d.
30	ascites	FIP	histopathology	effusion	IND	n.d.	n.d.
31	pleural effusion, fever	FIP	histopathology	effusion and serum	BLD	negative	n.d.
32	pleural effusion, icterus	FIP	histopathology	effusion and serum	FIPV	negative	M1058L
33	ascites, fever, icterus	FIP	histopathology	effusion	IND	n.d.	n.d.
34	ascites, icterus	FIP	histopathology	effusion	FIPV	n.d.	M1058L
35	ascites, icterus	FIP	histopathology	effusion and serum	BLD	BLD	n.d.
36	pleural effusion, fever, uveitis	FIP	histopathology	effusion	FIPV	n.d.	M1058L
37	pleural effusion, icterus, hyperglobulinemia	FIP	histopathology	effusion	BLD	n.d.	n.d.
38	ascites, icterus	FIP	histopathology plus immunohistochemistry	effusion	FIPV	n.d.	M1058L

*BLD* feline coronavirus present, but below limit of detection, *FIP* feline infectious peritonitis, *FIPV* feline infectious peritonitis virus, *IND* feline coronavirus present, but indeterminate sequence variations, *n.d.* not determined, *RT-PCR* reverse transcriptase polymerase chain reaction

Cats in the control group (*n* = 25) were suspected of having FIP (Table 2) based on the existence of one or more of the following signs consistent with FIP: effusion (*n* = 24), fever with ≤20,000 white blood cells/μL and ≤1000 band neutrophils/μL (*n* = 1), icterus (*n* = 2), or neurological signs (*n* = 1). Some of the included cats showed several of these signs. For all cats in the control group, a disease other than FIP was definitively diagnosed either at full post-mortem examination plus histopathology (*n* = 10), by histopathology of organ samples obtained post-mortem (*n* = 1), by bacterial culture and cytology diagnosing bacterial pleuritis (*n* = 2), by echocardiography diagnosing decompensated cardiac disease explaining pleural or abdominal effusion (*n* = 7), or by cytology diagnosing neoplasia (*n* = 5).

**Table 2:** Detailed information for cats of the control group

cat	symptoms leading to inclusion	diagnosis	method of confirmation of diagnosis	samples available	result of RT-PCR of effusion samples	result of RT-PCR of serum/plasma samples	detected mutation
1	pleural effusion	bacterial pleuritis	bacterial culture and cytology	effusion	negative	n.d.	n.d.
2	pleural effusion	chronic kidney disease, hypertensive encephalopathy; effusion most likely due to hypervolemia	full post-mortem examination including histopathology	effusion	FIPV	n.d.	M1058L
3	pleural effusion	decompensated cardiac disease	echocardiography	effusion	negative	n.d.	n.d.
4	pleural effusion	decompensated cardiac disease	echocardiography	effusion	negative	n.d.	n.d.
5	ascites	persistent foramen ovale	histopathology	effusion	negative	n.d.	n.d.
6	pleural effusion	decompensated cardiac disease	echocardiography	effusion	negative	n.d.	n.d.
7	pleural effusion	decompensated cardiac disease	echocardiography	effusion	negative	n.d.	n.d.
8	ascites	invasive pancreatic adenocarcinoma	histology of organ samples (obtained post-mortem)	effusion	negative	n.d.	n.d.
9	pleural effusion	decompensated cardiac disease	echocardiography	effusion	negative	n.d.	n.d.
10	pleural effusion	pulmonary adenocarcinoma	full post-mortem examination including histopathology	effusion	negative	n.d.	n.d.
11	pleural effusion	carcinoma	cytology	effusion	negative	n.d.	n.d.
12	ascites	carcinoma	cytology	effusion	negative	n.d.	n.d.
13	pleural effusion	lymphoma	cytology	effusion	negative	n.d.	n.d.
14	ascites	lymphoma	full post-mortem examination including histopathology	effusion and plasma	negative	negative	n.d.
15	pleural effusion	decompensated cardiac disease	echocardiography	effusion	negative	n.d.	n.d.
16	pleural effusion, neurological signs	carcinoma	full post-mortem examination including histopathology	effusion and plasma	negative	negative	n.d.
17	pleural effusion	chronic cardiomyopathy	full post-mortem examination including histopathology	effusion	negative	n.d.	n.d.
18	fever, icterus	cholangiohepatitis	full post-mortem examination including histopathology	plasma	n.d.	negative	n.d.
19	pleural effusion	pulmonary adenocarcinoma	full post-mortem examination including histopathology	effusion	negative	n.d.	n.d.
20	pleural effusion	lymphoma	full post-mortem examination including histopathology	effusion	negative	n.d.	n.d.
21	pleural effusion and ascites	malignant round cell tumor	cytology	effusion	BLD	n.d.	n.d.
22	pleural effusion	lymphoma	cytology	effusion	BLD	n.d.	n.d.
23	pleural effusion	decompensated cardiac disease	echocardiography	effusion	negative	n.d.	n.d.
24	pleural effusion	bacterial pleuritis	bacterial culture and cytology	effusion	negative	n.d.	n.d.
25	ascites	cholangiocarcinoma	full post-mortem examination including histopathology	effusion	negative	n.d.	n.d.

*BLD* feline coronavirus present, but below limit of detection, *FIPV* feline infectious peritonitis virus, *n.d.* not determined, *RT-PCR* reverse transcriptase polymerase chain reaction

### Samples

In total, 59 effusion samples and 17 serum/plasma samples were collected between 2009 and 2014. Effusion fluids (34 ascites, 25 pleural effusions) of 43 cats (25 with FIP, 18 controls) were stored at −80 °C and effusion fluids of 16 cats (ten with FIP, six controls) were stored at −20 °C. Of twelve of the cats (nine with FIP, three controls) for which blood was available, plasma was obtained and stored at −80 °C in 2 mL low temperature freezer vials (VWR International GmbH) until assayed. Of the remaining five cats (all of them had FIP) for which blood was available, serum was obtained and stored at −20 °C in 1.5 mL Eppendorf Safe-Lock microcentrifuge tubes (Eppendorf GmbH) until assayed.

All samples collected ante-mortem were originally obtained for diagnostic and, in the case of effusion, also for therapeutic purposes.

### Real-time RT-PCR

Real-time RT-PCR was performed blinded with regard to the final diagnosis.

Total nucleic acid was extracted from effusion and serum/plasma samples by QIAamp DNA Blood BioRobot MDx Kit on an automated Qiagen platform (QIAGEN GmbH, Hilden, Germany) according to the manufacturer instructions with slight modifications. In order to first detect FCoV and second to pathotype the FCoV strain, three real-time PCR assays were performed in parallel as singleplex reactions at a commercial reference laboratory (IDEXX Laboratories, Ludwigsburg, Germany): the first real-time PCR was based on the 7b gene [26] to quantify viral load, the other two real-time PCRs were targeting the M1058L and S1060A single nucleotide polymorphisms described before to correlate with the occurrence of the lethal FIPV genotype [15]. These two PCR tests allow typing of an FCoV strain based on the presence (FIPV) or absence (FECV) of one of two single nucleotide polymorphisms within the fusion peptide of the spike gene. Briefly, highly specific hydrolysis probes were designed to either detect the mutation at position 3174 or 3180 (corresponding to amino acid positions 1058 and 1060, M1058L and S1060A of reference sequence FJ938051 [15], respectively) or wildtype sequences by using an allelic discrimination approach using real-time PCR. Fluorescence intensities were used to calculate ratios of the probes detecting the mutation or the wildtype sequences. FIPV was assigned if the mutation probe exceeded a 2-fold higher fluorescence than the wildtype probe.

Real-time PCR was run with six quality controls (Table 3).

**Table 3:** Details of the six quality controls used in the real-time reverse transcriptase polymerase chain reaction (RT-PCR) assay

	quality controls	goal of quality controls
1	PCR positive controls (quantitatively¸ using synthetic DNA covering the real-time PCR target region (Integrated DNA Technologies IDT, Coralville, IA, USA))	functionality of PCR test protocols
2	PCR negative controls (PCR-grade nuclease free water)	absence of contamination in reagents
3	negative extraction controls (extraction positions filled with lysis solution and PCR-grade nuclease free water only)	absence of cross-contamination during the extraction process
4	RNA pre-analytical quality control targeting feline ssr rRNA (18S rRNA) gene complex	quality and integrity of the RNA as a measure of sample quality
5	a swab-based environmental contamination monitoring control	absence of contamination in laboratory
6	spike-in internal positive control (using lambda phage DNA)	absence of PCR inhibitory substances as a carryover from sample matrix

### Interpretation of real-time RT-PCR results

According to the outcome of the typing assay, there were six possible results of the real-time RT-PCR.

1. Pathotype FIPV: The mutated pathotype (containing either M1058L or S1060A) was detected in the sample.
2. Pathotype FECV: Feline enteric coronavirus without spike gene mutations was detected in the sample.
3. Mixed pathotype: A mixed population of FECV and FIPV was detected in the sample.
4. Below limit of detection (BLD): FCoV RNA viral load was low (below 1.5 million viral RNA equivalents per mL of sample). Owing to the insufficient number of viral RNA targets, pathotyping was not possible.
5. Indeterminate (IND): FCoV RNA viral load was high (above 1.5 million viral RNA equivalents per mL of sample), but pathotyping was not possible due to the occurrence of an unknown FCoV strain (failed amplification) or infection with a serotype II FCoV strain.
6. Negative: No FCoV RNA was detected in the sample.

### Statistical evaluation

Sensitivity, specificity, positive predictive value (PPV), negative predictive value (NPV), and overall accuracy (sum of true positive and true negative test results divided by the total number of test results) were calculated using a four-field-chart. To quantify uncertainty, 95% confidence intervals (95% CI) were calculated. A sample containing a mixed pathotype was defined as a positive result. Samples typed as BLD or IND were defined as a negative result, as no pathotype could be determined.

## Results

The FIPV pathotype was detected in 25/59 effusion samples. Of these, 24 were from cats with FIP, but one effusion sample from a control cat was also positive for FIPV with mutation M1058L. Mutation M1058L was found in 23/25 FIPV samples. Mutation S1060A was found in none of the FIPV samples. A mixed pathotype of FIPV and FECV was detected in 2/59 effusion samples (all from cats with FIP). In 12/59 effusion samples, FCoV RNA was detected, but pathotyping was not possible (BLD or IND). The remaining 22/59 effusion samples did not contain FCoV RNA (Tables 1, 2, 4, and 5).

**Table 4:** Results of effusion samples (*n* = 59)

group	FIPV^a^ M1058L	FIPV^a^ S1060A	FECV^b^	mixed pathotype^a^	BLD^b^	IND^b^	negative^b^	total
FIP	22	0	0	2	7	3	1	35
controls	1	0	0	0	2	0	21	24
total	23	0	0	2	9	3	22	59

*BLD* feline coronavirus present, but below limit of detection, *FECV* feline enteric coronavirus, *FIP* feline infectious peritonitis, *FIPV* feline infectious peritonitis virus, *IND* feline coronavirus present, but indeterminate sequence variations

^a^Defined as positive for statistical analysis

^b^Defined as negative for statistical analysis

**Table 5:** Results of effusion samples^a^

	FIP	control	total
positive	24	1	25
negative	11	23	34
total	35	24	59

*FIP* feline infectious peritonitis

^a^BLD (feline coronavirus present, but below limit of detection) and IND (feline coronavirus present, but indeterminate sequence variations) were defined as negative for statistical analysis, as the pathotype could not be determined. A mixed pathotype of feline infectious peritonitis virus and feline enteric coronavirus was defined as positive, as the mutated pathotype was detected

Real-time RT-PCR was negative in 15/17 serum/plasma samples. In the remaining two serum/plasma samples (all from cats with FIP), FCoV RNA could be detected, but only in low concentrations (BLD). Therefore, the pathotype could not be determined. None of the serum/plasma samples of control cats contained FCoV RNA (Tables 1, 2, 6, and 7).

**Table 6:** Results of serum/plasma samples (*n* = 17)

group	FIPV^a^ M1058L	FIPV^a^ S1060A	FECV^b^	mixed pathotype^a^	BLD^b^	IND^b^	negative^b^	total
FIP	0	0	0	0	2	0	12	14
controls	0	0	0	0	0	0	3	3
total	0	0	0	0	2	0	15	17

*BLD* feline coronavirus present, but below limit of detection, *FECV* feline enteric coronavirus, *FIP* feline infectious peritonitis, *FIPV* feline infectious peritonitis virus, *IND* feline coronavirus present, but indeterminate sequence variations

^a^Defined as positive for statistical analysis

^b^Defined as negative for statistical analysis

**Table 7:** Results of serum/plasma samples^a^

	FIP	control	total
positive	0	0	0
negative	14	3	17
total	14	3	17

*FIP* feline infectious peritonitis

^a^BLD (feline coronavirus present, but below limit of detection) and IND (feline coronavirus present, but indeterminate sequence variations) were defined as negative for statistical analysis, as the pathotype could not be determined. A mixed pathotype of feline infectious peritonitis virus and feline enteric coronavirus was defined as positive, as the mutated pathotype was detected

Sensitivity, specificity, PPV, NPV, and overall accuracy are shown in Table 8.

**Table 8:** Sensitivity, specificity, positive and negative predictive value, and overall accuracy of the real-time RT-PCR

	effusion	serum/plasma
sensitivity % (95% CI)	68.6 (50.7–83.2)	0 (0–23.2)
specificity % (95% CI)	95.8 (78.9–99.9)	n.d.
NPV % (95% CI)	67.6 (49.5–82.6)	17.6 (3.8–43.4)
PPV % (95% CI)	96.0 (80.0–99.9)	n.d.
overall accuracy % (95% CI)	79.7 (67.2–89.0)	17.6 (3.8–43.4)
FIP prevalence %	59.3	82.4

*FIP* feline infectious peritonitis, *n.d.* not determined, *NPV* negative predictive value, *PPV* positive predictive value, *RT-PCR* reverse transcriptase polymerase chain reaction, *95% CI* 95% confidence interval

## Discussion

This study evaluated the use of a new diagnostic test which is able to distinguish FIPV from FECV pathotypes in the diagnosis of FIP based on the presence of mutation M1058L or S1060A in the FCoV spike protein.

In a lethal disease like FIP, specificity of a diagnostic test is more important than sensitivity, because it helps to prevent euthanasia of cats misdiagnosed with FIP. Specificity of the real-time RT-PCR in effusion was 95.8%. The FIPV pathotype (M1058L) was found in an effusion sample from one control cat that had chronic kidney disease. There are several reasons that could explain this positive result. First, FCoV spike protein mutations M1058L and S1060A have previously been discussed as being a marker for the systemic spread of the virus rather than for the FIP phenotype, since they could also be found in tissue samples of healthy cats infected with FCoV [14]. If this was correct, then it would be possible that the cat was infected with a “benign” FCoV that spread systemically and therefore exhibited mutation M1058L. Second, full post-mortem examination including histopathology was performed in the cat and did not reveal any typical changes indicative of FIP. Nevertheless, it cannot be excluded that the cat suffered from early-stage FIP in addition to chronic kidney disease, but histopathological changes of FIP were still absent. Finally, it is possible that the result was a true false positive due to a methodological error.

The effusion samples of two control cats contained FCoV RNA but the pathotype could not be determined due to a low virus load (BLD). If a PCR had been used that was not able to differentiate pathotypes, these cats would have falsely been diagnosed as having FIP. This fact emphasizes that the detection of any FCoV in effusion is not accurate enough to establish the diagnosis FIP. It has been shown previously that FECV can circulate systemically in blood monocytes during initial infection [12].

A moderate sensitivity of 68.6% was found in effusion in the present study. This is comparable to or even lower than sensitivities reported in recent studies (65–89%) for different RT-PCR assays of effusions [22, 23, 27]. Most of these earlier studies determined the sensitivity of a RT-PCR that did not distinguish the two FCoV pathotypes [22, 23]. In contrast, the present study was designed to allow pathotyping of FCoV. In order to prevent false positive real-time RT-PCR results arising from the detection of rare random spike gene mutations, the degree of fluorescence for the reported pathotype needed to exceed twice that of the other pathotype. Therefore, even FCoV-positive samples were regarded as negative for the calculation of sensitivity if they did not allow definitive determination of either FIPV or FECV. Three of the effusion samples from cats with FIP typed as IND (high viral load but pathotyping was not possible) and therefore were considered negative for the calculation of sensitivity despite a high viral load. Additionally, in two of the serum/plasma and seven of the effusion samples from cats with FIP, FCoV RNA was detected, but the concentration was too low to allow pathotyping and therefore, these samples also were considered negative for the calculation of sensitivity. If sensitivity of the real-time RT-PCR had only been calculated for the detection of FCoV in general in the study population, then sensitivity would have been much better (97.1% for effusion and 14.3% for serum/plasma).

The FIPV pathotype was detected in the majority (24/34, 71%) of FCoV-positive effusion samples from cats with FIP. Substitution M1058L was found in 22/34 (65%), substitution S1060A in 0/34. These results are quite similar to a recent study detecting M1058L in 65% and S1060A in 6% of FCoV-positive effusions from cats with FIP [23]. Two of the effusion samples of cats with FIP were typed as mixed pathotype, meaning that populations of FECV and FIPV were present in the cat at the same time. It is likely that these cats were in an early stage during the transition of FECV to FIPV. Additionally, it is conceivable that these cats with FIP were superinfected with an FECV, as described previously [28, 29], and that their effusion samples were tested positive for both pathotypes due to leakage of FECV into the effusion.

As stated before, three of the effusion samples of cats with FIP typed as IND (high virus load but pathotyping was not possible). The reason for this might be the existence of unknown spike gene sequence variations in the sample, which are not recognized by the current primer set. Since the spike gene assay is specific for serotype I FCoV, infection with a serotype II FCoV also could cause typing as IND. Cats with FIP have been shown to exhibit higher viral loads than healthy FECV-infected cats [30] and therefore, if a sample is typed as IND, it is likely that the cat has FIP. Possibly, these cats exhibited alternative mutations in other parts of their genome that are characteristic for the development of the FIPV genotype. The 3c gene and other regions in the S1 and S2 domains of the spike gene have been identified as other potential sites for mutation(s) involved in FIP pathogenesis [16, 28, 29, 31, 32]. For example, variations in a furin cleavage site in the region between receptor-binding (S1) and fusion (S2) domains of the spike gene were detected when comparing FECV and FIPV sequences [16]. Another study compared FCoV from FIP lesions with FCoV from the feces of healthy cats and identified a consistent substitution of isoleucine with threonine at position 1108 of the spike protein in cats with FIP [32]. Additionally, mutations of the 3c gene might contribute to FIP pathogenesis. Mutations in this gene were observed in the majority of FIPV, whereas an intact 3c gene was detected in most FECV, suggesting that the 3c gene also plays a role in the pathogenesis of FIP [28, 29, 32, 33]. It should be considered to retest cats with effusion samples typed as BLD or IND in order to increase the possibility of correctly identifying the FIPV or FECV pathotype. Additionally, an effusion sample typed as IND should at least raise a strong suspicion of FIP, especially if other clinical or laboratory parameters are indicative of FIP.

Sensitivity in serum/plasma was low, confirming recent studies [22, 34]. A low concentration of FCoV RNA was detected in the serum/plasma of two cats with FIP, but the low virus load did not allow pathotype determination. Thus, sensitivity of the real-time RT-PCR in serum/plasma was 0%. This is in contrast to results of previous studies evaluating different RT-PCR assays and reporting sensitivities of 53–87% using serum, plasma, or whole blood [24, 25, 35, 36]. Nevertheless, in regard of the findings of a recent study, a low sensitivity in blood was expected, as FCoV RNA could not be detected in the whole blood, plasma, or white cell fraction of cats with experimentally induced FIP at any stage of disease. In the cats with FIP, viremia was either non-existent, or virus load was below the detection limit [34]. It is also likely that in the present study the majority of cats with FIP either were not viremic or that FCoV RNA levels were below detection limit of the real-time RT-PCR. It could be argued that sensitivity would have been better when investigating whole blood, as FIPV replication is restricted to macrophages [34, 37, 38]. However, real-time RT-PCR of serum and peripheral blood mononuclear cells (PBMC) has been compared and both showed rather low sensitivities, even though the sensitivity of PBMC (31.6%) was slightly better than that of serum (23.1%) [22]. In general, viral load in effusion is much higher than in blood [34].

One limitation of the present study was the inclusion criterion for some of the control cats. Histopathology could not be performed in all cats and confirmation of diagnosis was therefore achieved ante-mortem in 14 of the 25 control cats. Consequently, it cannot be totally excluded that some of these cats suffered from FIP in addition to their diagnosed diseases. Nevertheless, this seems rather unlikely, as real-time RT-PCR was false positive only in one of the control cats and in this specific cat, histopathology had been performed. A second limitation of the present study is the fact that in some of the cats, only one sample type (effusion or serum/plasma) was available and overall, the number of available serum/plasma samples was rather low.

## Conclusions

This study evaluated a discriminating real-time RT-PCR using effusion and/or serum/plasma in the diagnosis of FIP. The results indicate that the detection of the FIPV pathotype with substitution M1058L is very specific for the FIP phenotype and can be a useful tool in the diagnosis of FIP. Nevertheless, substitution M1058L was also detected in one control cat without FIP. As none of the FIPV-positive effusion samples contained substitution S1060A, it is considered a weak discriminatory factor for the diagnosis of FIP. The fact that in two other control cats FCoV was detected, even though the pathotype could not be determined, shows that FCoV can cause viremia and therefore, traditional non-discriminating RT-PCR is not sufficient to definitively diagnose FIP. Discriminative RT-PCR should be performed in order to minimize the risk of euthanasia of cats suffering from different diseases. The use of serum/plasma is not recommended owing to the low viral load in blood.

## Abbreviations

95% CI: 95% confidence interval
BLD: Below limit of detection
FCoV: Feline coronavirus
FECV: Feline enteric coronavirus
FIP: Feline infectious peritonitis
FIPV: Feline infectious peritonitis virus
IHC: Immunohistochemistry
IND: Indeterminate
NPV: Negative predictive value
PBMC: Peripheral blood mononuclear cells
PPV: Positive predictive value
RT-PCR: Reverse transcriptase polymerase chain reaction

## References

[1] Rottier PJ, Nakamura K, Schellen P, Volders H, Haijema BJ (2005). Acquisition of macrophage tropism during the pathogenesis of feline infectious peritonitis is determined by mutations in the feline coronavirus spike protein. J Virol.

[2] Poland AM, Vennema H, Foley JE, Pedersen NC (1996). Two related strains of feline infectious peritonitis virus isolated from immunocompromised cats infected with a feline enteric coronavirus. J Clin Microbiol.

[3] Vennema H, Poland A, Foley J, Pedersen NC (1998). Feline infectious peritonitis viruses arise by mutation from endemic feline enteric coronaviruses. Virology.

[4] Pedersen NC, Boyle JF, Floyd K, Fudge A, Barker J (1981). An enteric coronavirus infection of cats and its relationship to feline infectious peritonitis. Am J Vet Res.

[5] Pedersen NC (1976). Serologic studies of naturally occurring feline infectious peritonitis. Am J Vet Res.

[6] Horzinek M, Osterhaus A (1978). Feline infectious peritonitis: a coronavirus disease of cats. Small Anim Pract.

[7] Addie DD (2000). Clustering of feline coronaviruses in multicat households. Vet J.

[8] Cave TA, Golder MC, Simpson J, Addie DD (2004). Risk factors for feline coronavirus seropositivity in cats relinquished to a UK rescue charity. J Feline Med Surg.

[9] Pedersen NC, Sato R, Foley JE, Poland AM (2004). Common virus infections in cats, before and after being placed in shelters, with emphasis on feline enteric coronavirus. J Feline Med Surg.

[10] Pedersen NC, Holzworth J (1987). Coronavirus diseases (coronavirus enteritis, feline infectious peritonitis). Diseases of the cat, medicine and surgery.

[11] Pedersen NC, Boyle JF, Floyd K (1981). Infection studies in kittens, using feline infectious peritonitis virus propagated in cell culture. Am J Vet Res.

[12] Kipar A, Meli ML, Baptiste KE, Bowker LJ, Lutz H (2010). Sites of feline coronavirus persistence in healthy cats. J Gen Virol.

[13] Dewerchin HL, Cornelissen E, Nauwynck HJ (2005). Replication of feline coronaviruses in peripheral blood monocytes. Arch Virol.

[14] Porter E, Tasker S, Day MJ, Harley R, Kipar A, Siddell SG (2014). Amino acid changes in the spike protein of feline coronavirus correlate with systemic spread of virus from the intestine and not with feline infectious peritonitis. Vet Res.

[15] Chang HW, Egberink HF, Halpin R, Spiro DJ, Rottier PJ (2012). Spike protein fusion peptide and feline coronavirus virulence. Emerg Infect Dis.

[16] Licitra BN, Millet JK, Regan AD, Hamilton BS, Rinaldi VD, Duhamel GE (2013). Mutation in spike protein cleavage site and pathogenesis of feline coronavirus. Emerg Infect Dis.

[17] Kipar A, Meli ML (2014). Feline infectious peritonitis: still an enigma?. Vet Pathol.

[18] Pedersen NC (2009). A review of feline infectious peritonitis virus infection: 1963–2008. J Feline Med Surg.

[19] Addie DD, Paltrinieri S, Pedersen NC (2004). Recommendations from workshops of the second international feline coronavirus/feline infectious peritonitis symposium. J Feline Med Surg.

[20] Giori L, Giordano A, Giudice C, Grieco V, Paltrinieri S (2011). Performances of different diagnostic tests for feline infectious peritonitis in challenging clinical cases. J Small Anim Pract.

[21] Doenges SJ, Weber K, Dorsch R, Fux R, Fischer A, Matiasek LA (2016). Detection of feline coronavirus in cerebrospinal fluid for diagnosis of feline infectious peritonitis in cats with and without neurological signs. J Feline Med Surg.

[22] Doenges SJ, Weber K, Dorsch R, Fux R, Hartmann K (2017). Comparison of real-time reverse transcriptase polymerase chain reaction of peripheral blood mononuclear cells, serum and cell-free body cavity effusion for the diagnosis of feline infectious peritonitis. J Feline Med Surg.

[23] Longstaff L, Porter E, Crossley VJ, Hayhow SE, Helps CR, Tasker S (2017). Feline coronavirus quantitative reverse transcriptase polymerase chain reaction on effusion samples in cats with and without feline infectious peritonitis. J Feline Med Surg.

[24] Herrewegh AA, de Groot RJ, Cepica A, Egberink HF, Horzinek MC, Rottier PJ (1995). Detection of feline coronavirus RNA in feces, tissues, and body fluids of naturally infected cats by reverse transcriptase PCR. J Clin Microbiol.

[25] Gunn-Moore DA, Gruffydd-Jones TJ, Harbour DA (1998). Detection of feline coronaviruses by culture and reverse transcriptase-polymerase chain reaction of blood samples from healthy cats and cats with clinical feline infectious peritonitis. Vet Microbiol.

[26] Gut M, Leutenegger CM, Huder JB, Pedersen NC, Lutz H (1999). One-tube fluorogenic reverse transcription-polymerase chain reaction for the quantitation of feline coronaviruses. J Virol Methods.

[27] Felten S, Weider K, Doenges S, Gruendl S, Matiasek K, Hermanns W (2017). Detection of feline coronavirus spike gene mutations as a tool to diagnose feline infectious peritonitis. J Feline Med Surg.

[28] Bank-Wolf BR, Stallkamp I, Wiese S, Moritz A, Tekes G, Thiel HJ (2014). Mutations of 3c and spike protein genes correlate with the occurrence of feline infectious peritonitis. Vet Microbiol.

[29] Chang HW, de Groot RJ, Egberink HF, Rottier PJ (2010). Feline infectious peritonitis: insights into feline coronavirus pathobiogenesis and epidemiology based on genetic analysis of the viral 3c gene. J Gen Virol.

[30] Kipar A, Baptiste K, Barth A, Reinacher M (2006). Natural FCoV infection: cats with FIP exhibit significantly higher viral loads than healthy infected cats. J Feline Med Surg.

[31] Pedersen NC, Liu H, Scarlett J, Leutenegger CM, Golovko L, Kennedy H (2012). Feline infectious peritonitis: role of the feline coronavirus 3c gene in intestinal tropism and pathogenicity based upon isolates from resident and adopted shelter cats. Virus Res.

[32] Lewis CS, Porter E, Matthews D, Kipar A, Tasker S, Helps CR (2015). Genotyping coronaviruses associated with feline infectious peritonitis. J Gen Virol.

[33] Pedersen NC, Liu H, Dodd KA, Pesavento PA (2009). Significance of coronavirus mutants in feces and diseased tissues of cats suffering from feline infectious peritonitis. Viruses.

[34] Pedersen NC, Eckstrand C, Liu H, Leutenegger C, Murphy B (2015). Levels of feline infectious peritonitis virus in blood, effusions, and various tissues and the role of lymphopenia in disease outcome following experimental infection. Vet Microbiol.

[35] Hartmann K, Binder C, Hirschberger J, Cole D, Reinacher M, Schroo S (2003). Comparison of different tests to diagnose feline infectious peritonitis. J Vet Intern Med.

[36] Egberink HF, Herrewegh AP, Schuurman NM, van der Linde-Sipman JS, Horzinek MC, de Groot RJ (1995). FIP, easy to diagnose?. Vet Q.

[37] Weiss RC, Scott FW (1981). Pathogenesis of feline infectious peritonitis: nature and development of viremia. Am J Vet Res.

[38] Kipar A, May H, Menger S, Weber M, Leukert W, Reinacher M (2005). Morphologic features and development of granulomatous vasculitis in feline infectious peritonitis. Vet Pathol.

